# Matrix Effectors in the Pathogenesis of Keratinocyte-Derived Carcinomas

**DOI:** 10.3389/fmed.2022.879500

**Published:** 2022-04-29

**Authors:** Rafaela-Maria Kavasi, Monica Neagu, Carolina Constantin, Adriana Munteanu, Mihaela Surcel, Aristidis Tsatsakis, George N. Tzanakakis, Dragana Nikitovic

**Affiliations:** ^1^Laboratory of Histology-Embryology, Medical School, University of Crete, Heraklion, Greece; ^2^Immunology Laboratory, Victor Babes National Institute of Pathology, Bucharest, Romania; ^3^Colentina Hospital, Bucharest, Romania; ^4^Doctoral School, University of Bucharest, Bucharest, Romania; ^5^Forensic Science Department, Medical School, University of Crete, Heraklion, Greece

**Keywords:** extracellular matrix, hyaluronan, collagen, proteoglycans, metalloproteinases, skin

## Abstract

Basal cell carcinoma (BCC) and cutaneous squamous cell carcinoma (cSCC), referred to as keratinocyte carcinomas, are skin cancer with the highest incidence. BCCs, rarely metastasize; whereas, though generally not characterized by high lethality, approximately 2–4% of primary cSCCs metastasize with patients exhibiting poor prognosis. The extracellular matrix (ECM) serves as a scaffold that provides structural and biological support to cells in all human tissues. The main components of the ECM, including fibrillar proteins, proteoglycans (PGs), glycosaminoglycans (GAGs), and adhesion proteins such as fibronectin, are secreted by the cells in a tissue-specific manner, critical for the proper function of each organ. The skin compartmentalization to the epidermis and dermis compartments is based on a basement membrane (BM), a highly specialized network of ECM proteins that separate and unify the two compartments. The stiffness and assembly of BM and tensile forces affect tumor progenitors' invasion at the stratified epithelium's stromal border. Likewise, the mechanical properties of the stroma, e.g., stiffness, are directly correlated to the pathogenesis of the keratinocyte carcinomas. Since the ECM is a pool for various growth factors, cytokines, and chemokines, its' intense remodeling in the aberrant cancer tissue milieu affects biological functions, such as angiogenesis, adhesion, proliferation, or cell motility by regulating specific signaling pathways. This review discusses the structural and functional modulations of the keratinocyte carcinoma microenvironment. Furthermore, we debate how ECM remodeling affects the pathogenesis of these skin cancers.

## Introduction

“Keratinocyte carcinomas” refers to non-melanoma skin cancers (NMSCs), and the term usually defines basal cell carcinoma (BCC) and cutaneous squamous cell carcinoma (cSCC) (1). BCC is the most common malignancy worldwide, and cSCC is the most common metastatic skin cancer, the incidence of both increasing globally (2). BCC develops from basal cells, the deepest part of the epidermis layer, which moves upward to replenish the skin's barrier. cSCC arises from differentiated squamous keratinocytes populating the upper epidermis layers (3). Though generally not characterized by high lethality (1), approximately 2–4% of primary cSCCs metastasize. The prognosis of patients with metastatic cSCC is poor, highlighting the urgent need for novel therapeutic approaches and aggressive behavior biomarkers (4). In addition, in some cases, BCC attains metastasis potential and resistance to therapy due to smoothened (SMO) inhibitor resistance (5).

UV irradiation, the most common cause of NMSCs is correlated to the high mutation rate of cSCC (6). Moreover, additional factors, such as non-coding RNAs expression, are required to progress premalignant lesion, actinic keratosis (AK), to cSCC *in situ* (cSCCIS). In addition, UV-radiation triggering of oxidant production induces modulation of inflammatory signaling, resulting in immune suppression (7). Indeed, UV-radiation enhances transforming growth factor-β1 (TGF-β1) upregulation resulting in angiogenesis, inflammation, cancer-associated fibroblasts generation, and immune suppression, thus facilitating cancer survival, immune evasion, and finally metastasis (8). Moreover, several skin pathologic conditions such as recessive dystrophic epidermolysis bullosa (RDEB) (9), severe burns, or chronic ulcers may favor NMSC development (2). Both males and females are affected equally, while incidence increases with age.

The extracellular matrix (ECM) serves as a scaffold that provides structural and biological support to cells in all human tissues. The main components of the ECM, including fibrillar proteins proteoglycans (PGs), glycosaminoglycans (GAGs), and adhesion proteins such as fibronectin, are secreted by the cells in a tissue-specific manner, critical for the proper function of each organ (10). The ECM is classified into the pericellular, interstitial, and highly organized basement membranes (BM) (10).

Intriguingly, the biomolecules that constitute the ECM have attained structural and physicochemical properties that specifically facilitate the execution of their tissue-related biological functions (11). Thus, the abundant collagen fibers determine tissue's stiffness and mechanical strength. Furthermore, the heteropolysaccharides, GAGs, form molecular bridges between collagen fibrils to affect ECM viscoelasticity and contribute to tissues' compressive stiffness. Indeed, alterations in the orientation, density, crosslinking, and interactions of fibers induce aberrant matrix stiffness correlated to malignant tissue transformation (12).

Likewise, the ECM is a pool for various growth factors, cytokines, and chemokines (11). Therefore, the intense remodeling of the ECM in the aberrant cancer tissue milieu (12) affects biological functions such as angiogenesis, adhesion, proliferation, or cell motility by regulating specific signaling pathways (13, 14). In addition, the remodeling of ECM and tumor microenvironment facilitates the progression from premalignant forms such as actinic keratosis to invasive and metastatic cSCC (15).

### Multilayer Epithelium Mechanics in NMSCs

Mammalian skin comprises a multi-layered epithelium, e.g., the epidermis, dermis, and underlying connective tissue. The epidermis primarily consists of epithelial keratinocytes interconnected with enforced cell junctions (16). The skin compartmentalization to the epidermis and dermis compartments is based on a BM, a highly specialized network of ECM proteins that separate and unify the two compartments. The architecture of the BM is involved in tumor progression (17). Thus, BCCs, dependent on SmoM2 constitutive activators of Sonic hedgehog signaling (Shh), bud inward into the stroma, retaining their BM and rarely metastasizing (18). In contrast, RAS/MAPK-dependent SCCs commence as bidirectional tissue folds and exhibit invasiveness (19). The generation of SmoM2 and Ras respective mutants show changes in the expression of collagen 4a1/2 (COL4a1/2), Nidogen 1 (Nid1), and Sparc BM components.

Furthermore, in SCC, the mechanical forces exerted by overlying differentiated cells are sufficiently strong to confer loss of membrane integrity. Therefore, the stiffness and assembly of BM and tensile forces affect tumor progenitors' invasion at the stratified epithelium's stromal border (5, 20). In a separate study, Nid1 and COL4 expressions were decreased around the tumor nest of SCC but increased around the nest of BCC, further supporting BM input to the carcinoma phenotype. The strong expression of Nid1 and COL4 by stromal cells surrounding BCC, in contrast to cSCC, may prevent BCC cells from degrading the BM and invading the dermis (21).

### Mechanical Remodeling of NMSCs Stroma

The dermis consists of an abundant ECM, into which blood vessels, skin appendages, fibroblasts, and other mesenchymal cells are embedded (16). Notably, the mechanical organization of the ECM surrounding NMKC cells significantly differs compared to normal skin ECM (22). In contrast to epithelial cells, mesenchymal cells exhibit extensive cell-matrix interactions, generating strong contractile forces with increased cytoskeletal activities (23). At the injury site, fibroblasts transform into highly contractile myofibroblasts, characterized by enhanced cytoskeleton allowing them to exert more pronounced mechanical force to the surrounding ECM, which enhances physiological goals such as wound closure but promotes carcinogenesis (23, 24).

**Figure 1:**
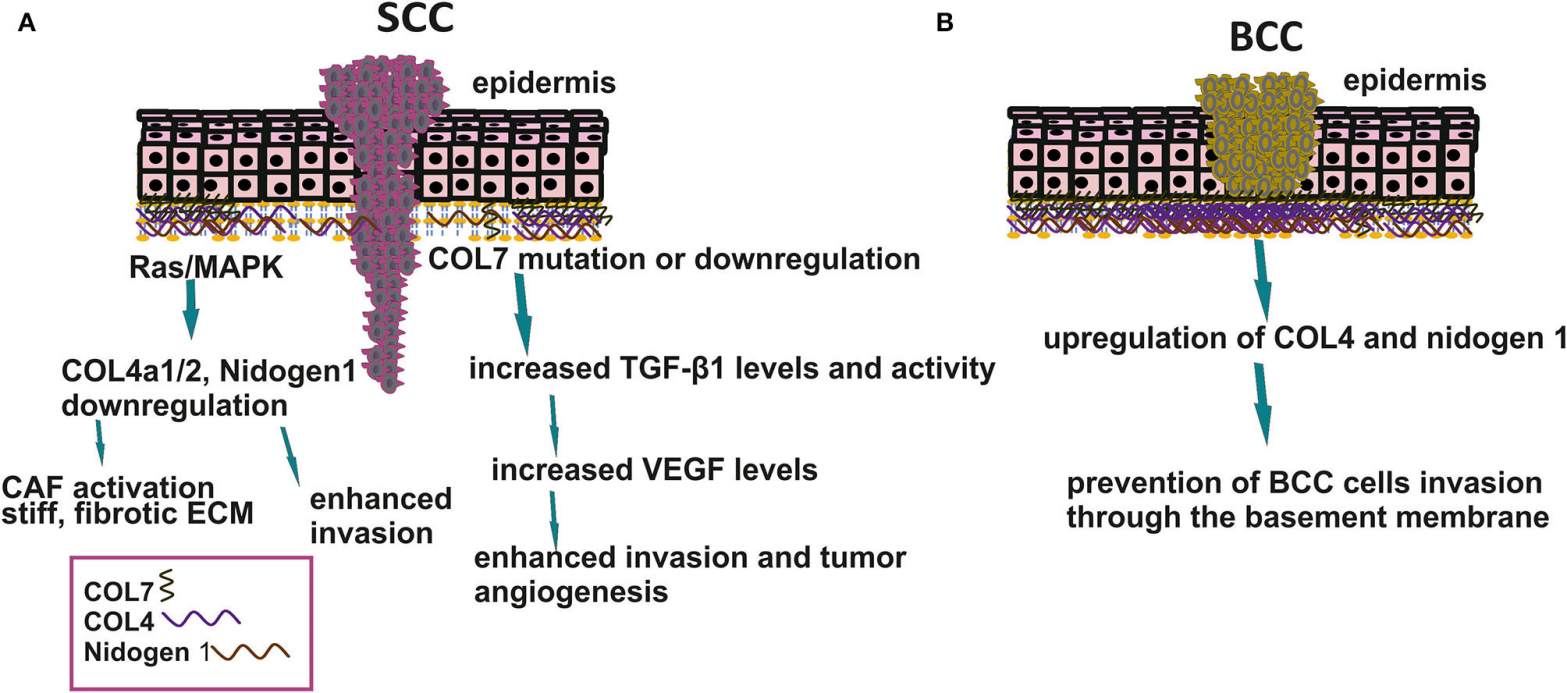
Basement membrane organization in SCC and BCC. **(A)** Downregulation in BM components enhances SCC cell invasion to the stroma affects matrix stiffness and tumor angiogenesis. **(B)** Upregulation of COL4 and nidogen 1 prevents BCC cells to metastasize.

Indeed, the stroma's mechanical properties, e.g., stiffness, are directly correlated to the pathogenesis of the NMKCs. Thus, patients with the highly disabling genodermatosis, the recessive dystrophic epidermolysis bullosa (RDEB), carry mutations in the COL7A1 gene encoding for type seven collagen, the main component of anchoring fibrils supporting cell binding to the BM. The majority of these patients progress to a highly aggressive type of cSCC (9). Thus, the resulting incompetence of the BM results in an inflammatory stroma profile where activated dermal fibroblasts promote cSCC development by creating a stiff, fibrotic ECM consisting of thick COL1 bundles, fibrinogen, and tenascin-C (25). Aspects of matrix-derived mechanotransduction signals are schematically depicted in Figure 1. Therefore, modifying the mechanotransduction signals could lead to novel therapeutic approaches for skin disease.

## ECM Effectors in NMSCs

### Hyaluronic Acid (HA) and Its Receptors

Hyaluronan (HA) is a significant component of skin ECM. This GAG is a linear, non-sulfated chain composed of repeating disaccharides of N-acetylglucosamine and glucuronic acid units (26). HA is synthesized by the three Hyaluronan Synthases (HAS1,2 and 3) isoforms as a high molecular weight product (HMWHA) (27). HASs are localized to the plasma membrane, and the newly synthesized HA chain is protruded to the ECM (26, 27). The degradation of HA to lower molecular weight HA (LMWHA) fragments is mediated by glycosidases, denominated hyaluronidases (HYALs).

HA exerts its biological functions *via* specific receptors, including the cluster of differentiation 44 (CD44) encoded by one gene but presenting several isoforms due to alternative splicing (28). HA also acts through the receptor for hyaluronan mediated motility (RHAMM), upregulated in cSCC (29). Toll-like receptor 4 (TLR4) is a transmembrane HA receptor whose signaling has been correlated to both skin inflammation (14) and cSCC progression (30). HA exerts strictly size-dependent biological effects. Thus, HMWHA exhibits anti-inflammatory, immunosuppressive, and anti-angiogenic properties, while LMWHA induces angiogenic, inflammatory, and tumorigenic impact (27).

HA metabolism is correlated to the pathogenesis of NMKCs. Thus, SSC tumors cells express alternative isoforms of CD44, associated with enhanced cell growth and cancer development (31). Furthermore, CD44 receptors can bind HA of a broad molecular weight range resulting in discrete downstream signaling. Thus, HMWHA through CD44/Rac-signaling in UV exposed mice promoted keratinocyte survival, normal differentiation, and DNA repair. In contrast, LMWHA-CD44 interactions facilitated a RhoA activation/NFκB/Stat-3 signaling/miR-21 production axis, leading to inflammation, abnormal keratinocyte proliferation, and SCC progression (31). Moreover, exposure of mice to chronic UV irradiation, the most common cause of cSCC, increased CD44 expression in mouse epidermis, while 20% of the exposed animals developed SCC tumors. Indeed, in this study, UV also upregulated HAS levels and HA synthesis, indicating the existence of a CD44/HA signaling axis (32). A similar upregulation of HAS2 and 3 was observed upon UV treatment in primary NHEK(F) cells. However, blocking interleukin-1β (Il-1β) activity minimized this effect, implying an interaction of HA metabolism with inflammatory signaling (33).

Interestingly, a combination of CD29 and CD44 was suggested to identify cancer stem-like cells (CSC) undergoing EMT in SCC. Thus, CD29high/CD44high expressing SCC cells were found to exhibit molecular markers of EMT, indicating that CSC-associated mechanisms contribute to the process of EMT (^34^). Moreover, in a separate study, a high CD44 phenotype in SCC is suggested to identify candidates for implementing targeted therapy aiming at CSCs (34).

Even though early studies did not detect a correlation between the CD44v isoform expression and SCC metastatic potential, recent reports show an enhanced expression of CD44v3-10, CD44v6-10, and CD44v8-10 isoforms co-expressed with CD44s and podoplanin in human SCC cell lines (35). Moreover, CD44 and the cell membrane glycoprotein, podoplanin, facilitated directional motility in epithelial cells and were shown to enhance SCC cell's directional migration.

Early studies implementation immunohistology show that BCCs displayed very low expression of CD44s, whereas the expression of CD44v6 exhibited a heterogeneous distribution pattern enhanced in the peripheral palisading tumor cells. In superficial BCCs, the labeling intensity for CD44v6 increased with the size of the tumor nests. These authors suggested that CD44v6 does not correlate with BCC metastatic potential, while the low CD44s is indicated as attenuating for BCCs metastasis (36). Moreover, in BCC, CD44 expression was reduced in metastasizing compared to non-metastasizing tumors. Together with increased Twist1 expression, this may suggest a critical EMT input in metastasizing tumors and characterize a metastatic phenotype (37). On the other hand, CD44 was one of the markers upregulated in BCC tumors compared to margins and controls (38). These data highlight the need for further study on the implications of the CD44/HA signaling axis and especially the determination of the expression of specific CD44 isoforms (39).

Notably, RHAMM has been related to keratinocyte motility, essential for wound healing and metastasis (40).

**Figure 2:**
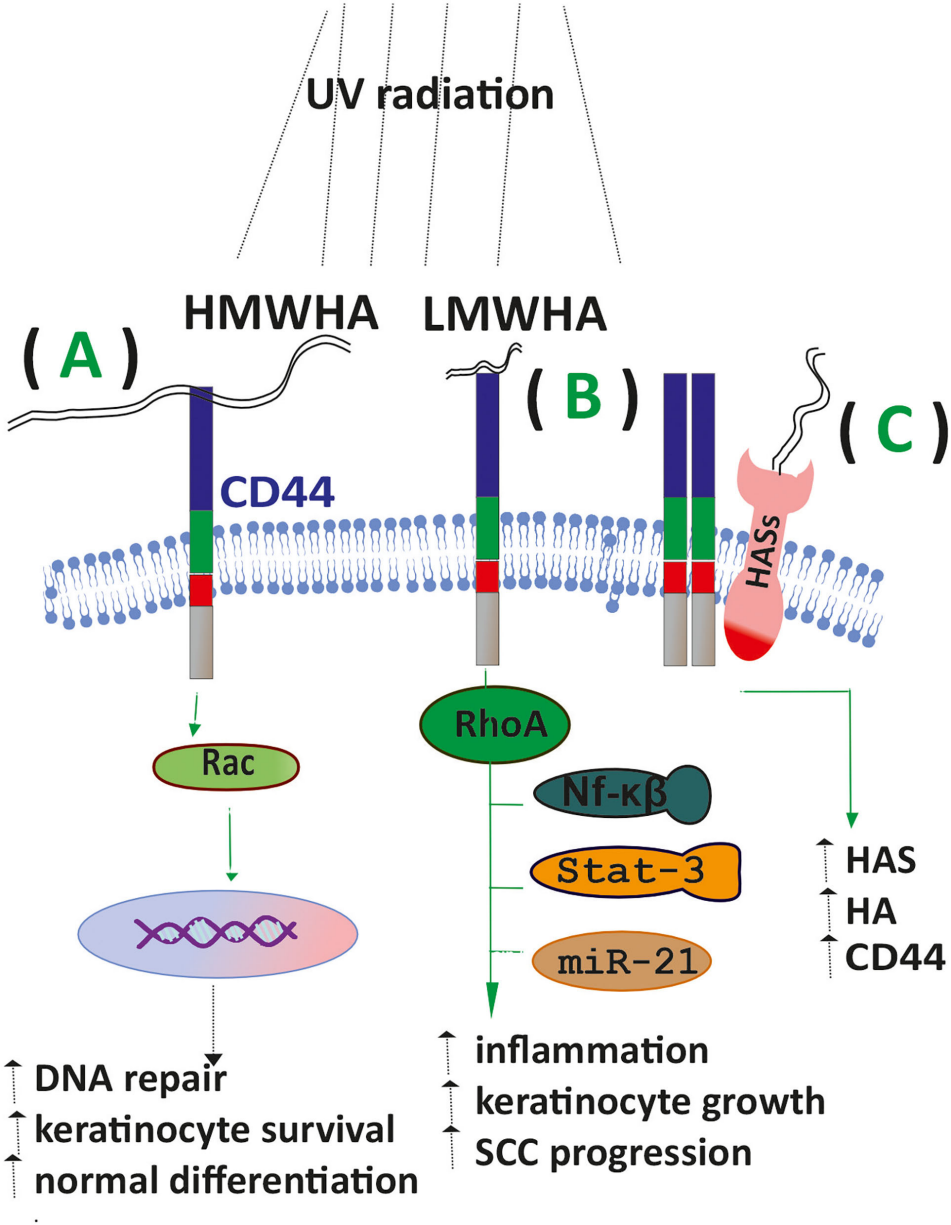
HA signaling affects SCC progression. **(A)** Upon exposure of keratinocytes to UV radiation, the binding of HMWHA to the CD44 receptor enhances DNA repair, keratinocyte survival, and normal differentiation. **(B)** LMWHA/CD44 binding in exposed keratinocytes promotes the inflammatory response, keratinocyte proliferation, and SCC progression. **(C)** Exposed keratinocytes show increased expression of HAS enzymes CD44 and increased HA deposition.

TLR4 is widely known for its implication in skin inflammatory responses, including allergic dermatitis (10). Moreover, in NMSC, it is overexpressed (41), whereas; in TLR4 knock-out mice, the progression of NKCS tumors was attenuated (42). The above data highlight the critical role of HA and its receptors in the mechanisms regulating the onset of NKCSs (Figure 2).

### Collagen Matrix Is Altered in NMSC

Collagens (COLs) are a primary group of ECM fiber proteins consisting of twenty-eight different types. COL participation is shown in various types of cancer, including NMSC (43). The skin content of collagen is reduced with age and is also affected by sunlight, especially UV exposure.

Indeed, in actinic keratosis, a loss of COL14 and 15 from the BM suggests that the ECM remodeling is an early sign of cSCC progression. Furthermore, the actinic keratosis stroma is negative for COL14 and 15, whose expression is evident later in the stroma of established cSCC tumors (44). Moreover, COL18 is detected in SCCs from grade one onwards, while normal keratinocytes in premalignant lesions were negative for this COL type (44).

Recessive dystrophic epidermolysis bullosa (RDEB) is a skin fragility disease caused by mutations that affect the function and/or the amount of COL 7, the primary component of anchoring fibrils. Hallmarks of RDEB are unremitting blistering and chronic wounds leading to tissue fibrosis and scarring. Nearly all patients with severe RDEB develop highly metastatic cSCC, the leading cause of death. Accumulating evidence from a murine RDEB model and human RDEB cells demonstrates that the lack of COL 7 also directly alters the wound healing process. Non-healing RDEB wounds are characterized by increased inflammation, high transforming growth factor-beta 1 (TGF-β1) levels and activity, and are heavily populated by fibroblast-derived, myofibroblasts responsible for enhanced fibrogenesis and matrix stiffness. In the next stage, fibroblasts are transformed into cancer-associated fibroblasts (CAF), contributing to cancer invasion and progression (45). Moreover, the *in vitro* downregulation of COL 7, which is crucial for the attachment of basal keratinocytes to the dermis, has been related to enhanced invasion in SCC (46). COL7 also suppresses TGF-β mediated downregulation of vascular endothelial growth factor (VEGF), responsible for SCC vascularization (47).

Collagen 17 (COL17), a dermo-epidermal junction structural component, is a type II hemidesmosomal transmembrane protein that stabilizes the hemidesmosome complex and basal keratinocyte binding to the BM (48). In addition to basal keratinocytes, the squamous keratinocytes around invasive tumor fronts, the adjacent tumor stroma, endothelium, and histiocytes exhibit upregulated COL7. Furthermore, aberrant shedding of COL7 ectodomain in SCC patients correlates to metastasis (49, 50). Indeed, the released COL17 endodomain enhances proliferation and survival, whereas the shedded ectodomain facilitates the invasiveness of SC cells (51).

An *in vitro* study with murine COL17a-/-keratinocytes revealed PI3K signaling activation. The upstream activators are the β4 integrin subunit of hemidesmosomal a6b4 integrin and the focal adhesion kinase (FAK), resulting in consequent Rac1 activation. This signaling pathway attenuated the ability of keratinocytes to migrate (52). Indeed, these authors suggest that COL17 facilitates keratinocyte adhesion and directed motility by interfering with the integrin-dependent PI3K activation and stabilizing lamellipodia in invasive cSCC. Moreover, the activity of integrin-β1 and COL17 expression at the intercellular site promotes contact following during the collective invasion of an SC cell population (53).

Reports mention the downregulation of total collagen deposition in NMSC patients (54, 55). Indeed, the total collagen levels are not significantly altered in a precancer state, suggesting that collagen downregulation could be considered a biomarker for cSCC occurrence. A possible explanation is the enhanced release of the collagen-degrading MMPs (55).

### Proteoglycans Mediate Keratinocyte-Tumor Progression

PGs are complex molecules whose protein core is glycosylated with one or more GAG chains (56). These versatile molecules regulate both the mechanical properties of tissues and act as key mediators of biological function. PGs are either secreted to the ECM or located to the cell membrane with the exception of serglycin, an intracellular PG (56). Due to their mediation of the cell/matrix interface PGs are involved in the pathogenesis of a multitude of cancer types (57–59).

Syndecans are a family of four transmembrane heparan sulfate PGs (HSPGs) exhibiting related structures. Indeed, all syndecan members consist of a variable N-terminal ectodomain, and highly conserved transmembrane and C-terminal cytoplasmic domains (60). Syndecans have been directly implicated in various tumor development (60, 61).

Syndecan-1 was shown to regulate intercellular and cell/matrix adhesion (60). Early studies on cutaneous biopsies of SCC detected a decrease of syndecan-1 expression with decreased adhesion of SCC cells suggesting that its' expression is inversely correlated to cSCC invasion into the dermis (62). This was supported by following studies showing that in invasive cSCC both syndecan-1 and cadherin E expressions were decreased with the loss of keratinocyte differentiation, especially in the acantholytic areas of tumors where interestingly these molecules were localized to the cytoplasm and not to the cell membrane (63), Indeed, syndecan-1 expression was identified to be discrete between keratoacanthoma and invasive SCC. Thus, syndecan-1 staining was diminished in invasive SCC compared to *in situ* SCC and keratoacanthoma (63). However, in a separate study, it was determined that syndecan-1 expression was decreased in BCC, cSCC, and metastatic human skin cancers compared to normal human skin (64). Indeed, Stepp et al. indicate that syndecan-1 is a significant factor in the early development and progression of skin cancers, suggesting that the expression of this PG could be utilized as a progression marker for cancerous skin lesions (64).

Interestingly, pioneer studies of the field demonstrated that basement membrane HSPGs and chondroitin sulfate PGs (CSPGs) were significantly decreased in cSCC compared to the normal epidermis (65).

Collectively, these data indicate that PGs, well established to regulate the cell to matrix adhesion as well as intercellular contacts important for epithelial cohesion, are important factors in keratinocyte carcinoma development and invasion to the dermis. Further studies defining their roles could improve the assessment of specific skin lesion prognoses.

### Matrix Metalloproteinases (MMPs)-Key Regulators of the Tumor Microenvironment

MMPs are zinc-containing endopeptidases that exhibit a wide range of substrate specificities, meditating, among others, the degradation of the different components of the ECM (66). However, the MMPs also act on membrane receptors, signaling molecules, and ligands modulating their activities and can thus be defined as cell signal regulators (67). Keratinocytes and dermal fibroblasts secrete these enzymes in response to various stimuli, including UV radiation, oxidative stress, and cytokines (68). For example, the exposure of keratinocytes to UV-radiation initiates critical cellular signaling pathways, including the nuclear factor-kappa beta (NF-kB), the mitogen-activated protein kinase (MAPK), the JAK/STAT (signal transduction and activation of transcription), and the nuclear factor erythroid 2-related factor 2 (Nrf2), critical regulators of inflammation and cancer (69). In continuation, exposed keratinocytes secrete active regulators that, in turn, activate the release of MMPs by dermal fibroblasts (70). The content of dermis ECM being remodeled by the MMPs facilitates an intricate balance that maintains the skin's homeostasis or its' response to injury. However, aberrant MMPs expression and activity in cancer tissues promote essential tumor-related functions (71).

Indeed, the invasion of tumor cells, a complex, multifaceted process, is initiated by MMPs degrading the BM and the surrounding ECM (72, 73). It is well established that the gelatinase MMP members, including MMP-2,−7, and−9, participate in BM degradation due to their capability to cleave key BM components such as COL4 (74–76). In addition, cancer-stroma interactions are essential at this point as MMP-2 is secreted chiefly by the surrounding fibroblast-like stromal cells and seldom by keratinocytes and BCC cells (77). Likewise, MMP-9 is secreted mainly by tumor-associated macrophages (TAMs), not BCC tumor cells (78). Interestingly, a higher number of macrophages, able to initiate BCC cells' MMP-9 secretion, utilizing the p38 MAPK/NF-kB/COX-2 cascade, was detected in aggressive forms of BCC compared to lees aggressive phenotypes (79). Furthermore, CAFs secrete MMP-1, MMP-3, MMP-7, MMP-9, and MMP-13, releasing ECM-bound growth factors such as VEGF supporting tumor angiogenesis (80).

Notably, MMP-1, MMP-7, MMP-9, MMP-13, and MMP-14 overexpression correlated to intensive remodeling of the BCC matrix was identified (81). Interestingly, a correlation between the MMP-2 and MMP-9 expression between the stroma and tumor suggests that tumor cells affect stroma MMPs deposition in BCC (82). However, in this study, no correlation between the MMP expression and the presence of the peritumoral cleft was shown (82). In chronic wounds, MMP-7, MMP-12, and MMP-13, but not MMP-1, MMP-3, MMP-8, MMP-9, and MMP-10 expressions, were identified as diagnostic clues for discerning SCCs from nonmalignant wounds (83).

A separate study suggested that MMP-1 has the highest COL1-degrading activity generating high molecular weight collagen fragments. There is an accumulation of these fragments as MMP-2 and MMP-9 have lower activities. The deposition of high molecular weight collagen fragments attenuates stromal fibroblasts' functions correlated with BCC progression (84). MMP-13 and MMP-1 also played a significant role in tumor progression of eyelid BCC in a clinical study, as immunoreaction positivity was identified in most patients (85). Likewise, MMP-11 was overexpressed in the biopsies of both cSCC and BCC patients compared to normal skin (86).

Interestingly the complement system seems to be involved in the MMP-dependent progression of non-melanoma skin cancers. Thus, the knock-out of the serine proteinase C1r, a complement system constituent, in cSCC led to these cells attenuating proliferation, migration, and invasion through collagen type I. Moreover, the knock-out of C1r downregulated the expression of MMP-1, MMP-13, MMP-10, and MMP-12 by cSCC cells in culture. In addition, knock-out of C1r decreased the expression of MMP-13, inhibited the invasion of cSCC, and decreased collagen cSCC xenografts (87). Therefore, the authors suggest that the complement facilitates SCC cells' invasion by enhancing MMPs' expression.

Intriguingly, in NMSC patients, antibodies against MMP-7 were detected. As these autoantibodies are being utilized as an early marker for other malignancies, it was suggested that they could also be a biomarker for NMSCs. Notably, the expression of MMP7 autoantibodies was not followed by changes in the MMP-7 mRNA levels in both BCC and cSCC patients (88). On the other hand, at the protein level, the expression of MMP-7 and CD44 was found to be inversely correlated in cSCC and BCC sections. Indeed, in well-differentiated SCC, the MMP-7 staining was weak, while CD44 expression was high. In poorly differentiated SCC, increased MMP-7 staining was identified while the expression of CD44 was weak (89).

MMPs expression is correlated to the progression and recurrence of these tumor types. cSCC patients' tumor tissues exhibited a notably higher level of MMP-13 compared to control tissues. In addition, these patients showed significantly increased MMP-13 levels compared to healthy controls. Moreover, patients with invasive SCC had increased serum MMP-13 levels compared to *in situ* cSCC patients (90). Therefore Wang et al. suggest that serum MMP-13 is a relevant diagnostic marker for cSCC (90). In cSCC and BCC biopsies, MMP-2 expression was correlated to the depth of invasion, whereas the expression of MMP-2, MMP-9, TIMP-1 with inflammation and microvessel density (91). Likewise, Gozdzialska et al. show that MMP-2 protein expression in the tumor stroma was higher in high-risk BCCs than low-risk BCCs, whereas MMP-2 deposition was enhanced in both the tumor tissue and stroma in SCC when compared to BCC (92). These data point to a crucial role of MMP-2 in skin cancer invasion (92).

#### MMPs Involvement in Immune-Related Processes in NMSCs

As already highlighted, the tumor microenvironment plays an essential role in the interplay between tumors and the elements of the immune system, mainly the immune-infiltrating cells and their immune molecules (93, 94). In recent years, as the gathered information regarding anti-tumoral immunity is piling up, the importance of immune-infiltrating cells in tumors (TIL), including skin cancers (95), is steadily increasing. A recent study that has analyzed data from various databases (e.g., The Cancer Genome Atlas, ONCOMINE) showed that immune cells' infiltration, MMPs expression, and tumor prognosis are associated in many types of cancers (96).

#### Immune Cells Infiltration in NMSCs

Although new investigation methods have recently entered the clinical management of NMSC patients (97), new insights into the local and circulatory immunity are needed (98). Several immune cell families infiltrate the tumor, out of which tumor-associated macrophages (TAM) and lymphocytes comprise the majority of immune cells. Notably, the relation between MMP14 expression and tumor-infiltrating lymphocytes (TILs) was investigated in several cancers. The findings indicate that MMPs, more specifically MMP14, can be a good prognosis, diagnosis, and treatment biomarker in various solid tumors (99).

It was shown, during SCC progression, that TAMs secrete several pro-carcinogenesis molecules such as endothelial vascular growth factor (VEGF)-C and MMPs, with a predominance of MMP9, MMP10, and MMP11. This array of TAM-related molecules encourages other inflammatory cells to enter the tumor and release cytokines that further promote tumor growth and angiogenesis (100, 101). Furthermore, a rich infiltrate with TAMs expressing predominantly M2 type induces lymph node metastasis in SCC (102); concomitantly high MMP expression is associated with advanced stages of SCC (103).

A recent *in vitro* cellular model study tested antibodies specific to MMP-1 conjugated to iron-gold bimetallic nanoparticles. In a cell line of human tongue squamous cell carcinoma (HSC-3), these antibodies triggered a high percentage of tumor cells death (104). Indeed, tumor cells attenuate the immune anti-tumoral action by MMP-2, MMP-9, MMP-13, and MMP-14 secretion, leading to T-cell activity hindrance (105).

In BCC, it was demonstrated that the tumor harbors immature dendritic cells with low potency in antigen presentation and an array of Th2-type immunosuppressive cytokines (77, 106). Earlier studies have shown that MMPs have various roles in the epidermal–mesenchymal transition processes and the immune response during the progression of BCC. Indeed, cocultivation of fibroblasts and melanoma cells has resulted in MMP-2 high overexpression, whereas; keratinocytes and BCC cells cocultivation led to a moderate upregulation of MMP-2 expression. However, in the absence of cellular contact, when only molecular communication was established between fibroblasts with BCC cells, a decrease in fibroblasts' MMP-2 expression was observed (78). These data suggest that cell-cell signaling is obligatory in regulating MMP-2 expression in this system.

In immunocompetent subjects, MMP-1, MMP-9, and TIMP-1 expression associated with stromal macrophages in BCC (107) and TILs comprise CD4 + T-helper cells and FoxP3 + T-regulatory (Tregs); these immune cell populations can also be found in the peritumoral inflammatory infiltrates. The proportion of these cellular immune populations is associated with the recurrence risk in this skin cancer. A high proportion of cytotoxic CD8+ T cells in TILs characterizes an antitumor response, while a lower ratio is associated with an increased risk of recurrence (108). An earlier study performed in BCC animal models has shown that tumor growth is promoted when TILs have low cutaneous macrophages and dendritic cells proportion (109). Additionally, an increased number of immunosuppressive Tregs is associated with an unfavorable outcome (110, 111), but it is still unknown if TILs in BCC come from skin resident cells or circulating lymphocytes (110).

MMPs are involved in the immune response flow in this immune tissue portrayal. Thus, MMPs can cleave an important T cellular receptor, such as IL-2Rα, and hence impede this effector cell proliferation. Moreover, MMP-11 can participate in the increased survival of BCC tumor cells even when NK cells release cytotoxic molecules (111). Cancer-associated fibroblasts (CAFs) are another essential cell population found in tumors. In BCC's stroma and the peritumoral area, CAFs can secrete an array of MMPs (e.g., MMP-1, MMP-2, MMP-3, MMP-9, MMP-11, MMP-13, MMP-14, MMP-19), all of them promoting ECM remodeling (105, 111) and favoring tumor escape from the anti-tumoral action of the immune cells (109).

Various immune-related surface molecules are involved in delivering an efficient anti-tumoral action. Programmed cell death-1 protein (PD-1) is a vital surface molecule from the CD28 superfamily highly involved in the anti-tumoral T cell activity (112). In BCCs, the expression of PD-1 and PDL-1 was found on tumor cells in 22% of cases and over 80% of TILs and TAMs (113). A recent study on esophageal SCC has shown that upregulation of several key molecules (CTLA-4, PD-1, PD-L1, TIM-3, LAG-3) are associated with MMP-13 expression and correlated to tumor progression and invasion (114).

Similarly, to BCC, in cSCC' TILs display specific patterns owing to the immune-suppressive tumor environment. This tumor environment induces decreased proliferation of TILs, impairs cytokine secretion, and hinders their capability to effectively kill tumor cells (115). cSCC has a predominantly Th2 type immune response, with low interferon (IFN) gamma (106) so that M2 type TAMs are activated, attracting new Tregs cellular populations and further sustaining the immune-suppressive milieu (116), processes correlated with an unfavorable prognosis (117). In laryngeal SCC, it was shown that tumor cells interact with ECM and that MMPs destroy the ECM scaffold to promote tumoral invasion (118).

MMP-9 is secreted by leukocytes and is associated with various tumor cells' aberrant behavior (119). In laryngeal SCC, MMP-9 expression is correlated with recurrence prognosis and poor outcome (120). A recent study showed that the expressions of GRK2, TRAF2, and MMP-9 in laryngeal SCC clinical samples were found significantly increased compared to normal tissues. These results proved that two immune-related signaling pathways cross-talk, TNF-α and PGE2. PGE2 increased the pathway TNF-α-TRAF2-MMP-9 signaling. Therefore TRAF2 and GRK2 co-expression induce proliferation, migration, and invasion of tumor cells, further promoting MMP-9 expression (121).

#### Circulatory MMPs and Immune-Related Markers in NMSC

Tumor-associated antigens (TAAs), discovered more than 20 years ago, are over-expressed in neoplastic transformed tissue and circulate in the peripheral blood (122). The immune system recognizes TAAs, as transformed antigens and synthesizes/secretes specific autoantibodies (123).

In the last ten years, serum autoantibodies against TAAs were evaluated in various cancers, but the first study focusing on NMSC's evaluation of TAA autoantibodies was published in 2021. In this study, autoantibodies against p53, MMP-7, and Hsp70 were significantly higher in NMSCs patients than normal subjects. Moreover, this panel of TAA autoantibodies can establish early diagnosis of NMSC (88).

A similar study has shown that cSCC patients display high serum MMP-13. After surgery, the MMP-13 level decreases and, when raising again, can predict an invasive SCC and lymph node metastasis; thus MMP-13 can represent a reliable biomarker for invasiveness and tumor progression monitoring (90).

Several years ago, in SCC, circulating immune cell populations were reported as altered compared to normal subjects; the proportion of circulatory sub-populations and their activation pathways deregulations were identified (83). Recently, we have shown that there are critical circulatory disturbances among immune cells in oral SCC. Hence, pre-surgery patients display significantly higher suppressive T lymphocytes and increased circulating NK (CD16+) cells than post-surgery levels. The results aid the immuno-inflammatory portrait of SCC (124).

**Figure 3:**
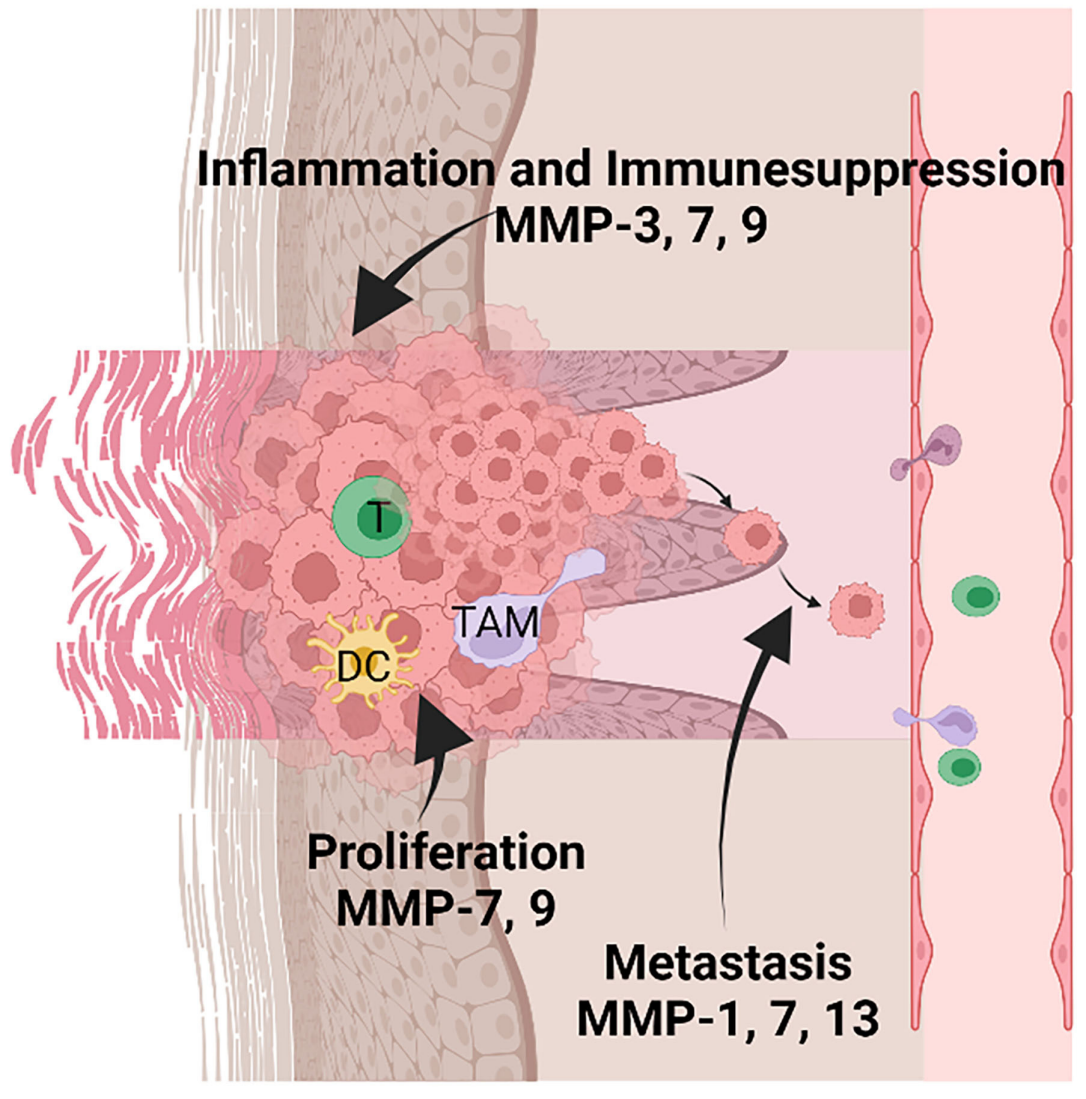
MMPs' involvement in inflammation, tumor proliferation, and metastasis in keratinocyte-derived tumors. Tumor-infiltrating immune cells are comprised of several populations such as T lymphocytes (T), dendritic cells (DC), and tumor-associated macrophages (TAM). MMP-3, 7, and 9 are mainly involved in sustaining the inflammatory microenvironment and suppressing the anti-tumoral action of the immune cells. In addition, MMP-7 and 9 sustain the proliferation of tumor cells; MMP-1, 7, 13 favor the metastatic processes by activating the invasion of tumor cells in other tissues. Created with BioRender.”

The novel, recent players that provide information regarding the inflammatory status of NMSCs, are the non-coding RNAs (nc-RNAs). These molecules have epigenetic regulatory action and are involved in skin cancers (125) as well as associated with the inflammatory processes related to skin's UV irradiation. Hence upon UVB radiation of the skin, it was shown that nc886 expression decreases, which drives uncontrolled PKR activity and increases in the MMP-9 expression and other inflammatory mediators production (e.g., type IV collagenase, COX-2). This recent finding indicates that ncRNAs could be a new therapeutic target in skin inflammation and carcinogenesis (126). The MMP's involvement in cSCCs progression is depicted in Figure 3.

#### Therapies Based on MMP Targeting

Immune checkpoint blockade therapy is already applied to manage several skin cancers (127). As molecules like CTLA4, PD-1, and PD-L1 are already therapy targets (128), improvements are sought to treat non-responsive patients (129). Hence, as previously shown, MMPs are associated with tumor microenvironment and infiltrating immune cells. Notably, recent studies have shown that inhibition of MMP2/9 can reduce tumor burden and improve survival, promoting anti-tumoral immunity. Upon inhibiting these MMPs, it was demonstrated that mRNA and protein levels of PD-L1 expression were reduced (130).

Epoxy-taglines are a novel class of compounds with anti-cancer properties to be used in skin tumors. The compound targets keratinocyte-derived tumors and promotes re-epithelialization after tumor tissue destruction. A recent *in vitro* study has shown that epoxy-tiglianes modulate MMP and cytokine gene expression along with other molecular processes that favor keratinocyte healing responses (131).

## Conclusion: Challenges and Developments

The changes in the structure/function of the ECM in NMSC are well documented. The ECM alterations affect both multilayer epithelium mechanics and the mechanical remodeling of the stroma. In addition, the changes in ECM components either by generating matrikines or releasing growth factors, cytokines, and other signaling mediators essentially affect all steps in these tumors' development and dissemination. Notably, increasing evidence demonstrates that matrix-associated molecules can be utilized as diagnosis/prognosis markers and therapy venues. Thus, CD44 and CD29 have been determined as cancer-stem cell markers (132) and a high CD44 phenotype in SCC is suggested to identify candidates for implementing targeted therapy (34). Likewise, MMP-9 is indicated as a marker of aggressive BCC forms (79). Matrix molecules such as MMP-13 (90) and MMP-7 (88) are identified as TAAs circulating in the peripheral blood. Recently, nc-RNAs have been determined as positive regulators of inflammatory mediators including MMP-9 (126) suggesting that these molecules could be a new therapeutic target in skin inflammation and carcinogenesis.

However, further studies need to be performed to correlate discrete ECM molecules' characteristics that can predict facets of tumor behavior regarding their aggressiveness and susceptibility to discrete therapeutic strategies. Moreover, systemic treatment may be indicated in the case of locally advanced or metastatic disease. Indeed, valid systemic therapy for keratinocyte carcinomas remains an unmet clinical need. Therefore, better defining the molecular mechanisms supporting tumor growth and development is crucial for the needed advances.

## Author Contributions

R-MK and DN conceived, designed the structure, and contributed to the writing of the paper. MN, CC, AM, and MS contributed to the writing of the paper and the preparation of figures. GT, AM, and DN edited the paper. All authors contributed to the article and approved the submitted version.

## Funding

The APC was funded by grant NASR, [PN 19.29.01.01] (50%) and by the Romanian Company Dialab Solutions (50%). Dialab Solutions was not involved in the study design, collection, analysis, interpretation of data, the writing of this article or the decision to submit it for publication. DN was partially funded by the Research Committee of the University of Crete (ELKE), grant number (KA:10393). Some research noted in text has been partly supported by the above grants.

## Conflict of Interest

The authors declare that the research was conducted in the absence of any commercial or financial relationships that could be construed as a potential conflict of interest.

## Publisher's Note

All claims expressed in this article are solely those of the authors and do not necessarily represent those of their affiliated organizations, or those of the publisher, the editors and the reviewers. Any product that may be evaluated in this article, or claim that may be made by its manufacturer, is not guaranteed or endorsed by the publisher.
